# Design, Characterization, and In Vitro Assays on Muscle Cells of Endocannabinoid-like Molecule Loaded Lipid Nanoparticles for a Therapeutic Anti-Inflammatory Approach to Sarcopenia

**DOI:** 10.3390/pharmaceutics14030648

**Published:** 2022-03-16

**Authors:** Eleonora Maretti, Susanna Molinari, Renata Battini, Cecilia Rustichelli, Eleonora Truzzi, Valentina Iannuccelli, Eliana Leo

**Affiliations:** Department of Life Sciences, University of Modena and Reggio Emilia, Via Campi 103, 41125 Modena, Italy; eleonora.maretti@unimore.it (E.M.); susanna.molinari@unimore.it (S.M.); renata.battini@unimore.it (R.B.); cecilia.rustichelli@unimore.it (C.R.); eleonora.truzzi@unimore.it (E.T.); valentina.iannuccelli@unimore.it (V.I.)

**Keywords:** C2C12 cells, solid lipid nanoparticles, SLN, palmitoylethanolamide, PEA, nanocarriers, muscle myoblast

## Abstract

Inflammatory processes play a key role in the pathogenesis of sarcopenia owing to their effects on the balance between muscle protein breakdown and synthesis. Palmitoylethanolamide (PEA), an endocannabinoid-like molecule, has been well documented for its anti-inflammatory properties, suggesting its possible beneficial use to counteract sarcopenia. The promising therapeutic effects of PEA are, however, impaired by its poor bioavailability. In order to overcome this limitation, the present study focused on the encapsulation of PEA in solid lipid nanoparticles (PEA-SLNs) in a perspective of a systemic administration. PEA-SLNs were characterized for their physico-chemical properties as well as cytotoxicity and cell internalization capacity on C2C12 myoblast cells. Their size was approximately 250 nm and the encapsulation efficiency reached 90%. Differential scanning calorimetry analyses demonstrated the amorphous state of PEA in the inner SLN matrix, which improved PEA dissolution, as observed in the in vitro assays. Despite the high internalization capacity observed with the flow cytometer (values between 85 and 94% after 14 h of incubation), the Nile Red labeled PEA-SLNs showed practically no toxicity towards myoblasts. Confocal analysis showed the presence of SLNs in the cytoplasm and not in the nucleus. These results suggest the potentiality provided by PEA-SLNs to obtain an innovative and side-effect-free tool in the medical treatment of sarcopenia.

## 1. Introduction

Sarcopenia is a syndrome characterized by progressive and generalized loss of skeletal muscle mass and strength combined with low physical performance, as ratified by the European Working Group on Sarcopenia in Older People [1]. It affects more than 50 million people worldwide and it is the most important cause of physical frailty, reducing the possibility of an independent life in older adults [2,3]. Sarcopenia could occur with normal aging and also be associated to rheumatologic disease frequently encountered in the elderly population, such as rheumatoid arthritis in women [4].

The current treatment or prevention for sarcopenia combines aerobic and resistance physical training associated with nutritional therapy (amino acids, proteins, vitamin D, polyunsaturated fatty acids). Exercise has been proven to produce the most effective results against muscle wasting; however, immobile patients and the inter-individual differences in physical capacity restrict the ability of exercise to counteract sarcopenia [5]. Drug therapies under consideration are based on the etiological and biological mechanisms of sarcopenia, especially on modulation of receptor signaling, even if they are not yet fully understood [5,6]. In the last few years, drug delivery systems, mainly based on nanoparticles and muscle-targeting delivery systems, have been also developed for the systemic treatment of sarcopenia and other muscle disorders [7,8].

One of the most accredited causes of sarcopenia seems to be linked to the reduced function of satellite cells, muscle resident stem cells which are responsible for muscle growth and repair, as well as to myocyte apoptosis, both mediated by the increased inflammation and pro-inflammation cytokines, particularly IL-6 and TNF-α [5,9]. Furthermore, recent findings about the pathophysiology of sarcopenia have attributed an important role to inflammatory processes owing to their effects on muscle protein breakdown and synthesis [8,10,11,12]. The oral administration of the usual non-steroidal anti-inflammatory drugs (NSAIDs) has been shown to improve muscle protein metabolism in low-grade inflammation diseases that act upon cyclooxygenase (COX)-inhibiting pathways [10,13,14]. Conventional NSAIDs therapy, however, is not recommended for sarcopenia treatment owing to its side effects in the elderly, especially considering the daily dosing and the long-term therapy. Other safer anti-inflammatory compounds have been poorly investigated, with the exception of vitamin D, curcumin, and polyunsaturated fatty acids [10,15].

Among natural compounds, palmitoylethanolamide (PEA), an endogenous endocannabinoid-like molecule, has been well documented for its anti-inflammatory properties and indicated for the treatment of several diseases by clinical data [16,17]. PEA anti-inflammatory action involves inhibition of the major pro-inflammatory cytokines, including TNF-α and interleukins, as well as reduction of COX-2 expression and/or prostaglandin release [16,18,19,20,21]. Unlike what happens with NSAIDs, PEA specifically targets COX-2, determining effects on intramuscular prostaglandin2 levels and satellite cell activity with concomitant lower incidence of adverse effects [18,22,23,24]. Both interleukins and COX-2 are expressed in response to inflammation by human myoblasts. However, muscle diseases including sarcopenia have never been considered as investigational targets of PEA; despite its potential pharmacological properties, PEA is currently available in marketed veterinary, nutraceutical, and cosmetic products. With respect to pharmaceutics, preclinical and clinical studies have provided evidence of conceivable usefulness of PEA as anti-inflammatory agent, enabling also to decrease doses of co-administered NSAIDs in long-term therapy for several pathologies. In particular, these studies have involved PEA as micronized or ultra-micronized form to increase its solubility and oral bioavailability [25,26,27].

Another approach to improve features regarding bioavailability and cellular targeting may involve nanotechnology. Nanoparticles are recognized for their ability to overcome biological barriers, produce sustained drug release depot when administered intramuscularly or subcutaneously, improve cell affinity, and gradually release the embedded drugs providing increased drug bioavailability. Moreover, nanoparticles administered intravenously provide a complete drug bioavailability, with rapid response even at low doses and a subsequent distribution in muscles [28,29].

However, to our knowledge, the few studies dealing with nano-encapsulated PEA have focused on skin and ocular applications [30,31].

On this basis, the present research aims to envisage the development of PEA-loaded solid lipid nanoparticles (SLNs) from the perspective of an anti-inflammatory activity able to regulate the age-related sarcopenia. Herein, PEA-loaded SLNs were characterized in terms of physico-chemical properties as well as cytotoxicity and capacity to be internalized by C2C12 myoblast cells.

## 2. Materials and Methods

### 2.1. Materials

PEA used for the preparation of all SLN samples was a gift from Innexus Nutraceuticals (Nijmegen, The Netherlands). Stearic acid, Pluronic F68, sorbitan trioleate (Span 85), and Nile Red (NR) were from Sigma-Aldrich (Milan, Italy); cholesteryl stearate was from Fluka Chemie AG (Buchs, Switzerland); and sodium cholate was obtained from Alfa Aesar (Karlsruhe, Germany). High purity water was obtained from MilliQ system (Millipore, MA, USA). The C2C12 cell lines of mouse myoblasts used for the cytotoxicity and cell internalization investigations were originally obtained by M. Buckingham (Institut Pasteur, Paris, France). Dulbecco’s Modification of Eagle’s Medium (DMEM) with high glucose, sodium pyruvate, L-glutamine, fetal bovine serum (FBS), penicillin-streptomycin (P/S), pH 7.4 phosphate buffer saline (PBS), and trypsin were purchased from EuroClone (Milan, Italy), while fetal bovine serum (FBS) (Gibco) was from Thermo Fisher Scientific (Monza, Italy). All the other chemicals were of analytical grade.

### 2.2. PEA Solubility in the Lipids

The solubility of PEA in the selected lipids (namely, stearic acid and cholesteryl stearate) was determined at the temperature of 85 °C under 200 rpm magnetic stirring [32]. Exactly weighted amounts of PEA were gradually added to known amounts of stearic acid or cholesteryl stearate and a mixture thereof with the same ratio used for SLNs preparation; the progressive additions were made at 30 min intervals until concentrations exceeded saturation solubility. Maximum amounts of solute, averaged on three determinations, were considered as the solubility values.

### 2.3. SLNs Preparation

SLNs loaded with PEA (PEA-SLNs) were prepared by the melt emulsification technique [33,34,35,36]. PEA (15 mg) was dissolved in a stearic acid (40 mg)/cholesteryl stearate (30 mg) blend containing Span 85 (30 mg) melted at a temperature of 85 °C. Then, the aqueous phase (5 mL Milli-Q water) containing 0.3% Pluronic F68 was heated at the same temperature and added to the lipid phase. The emulsification was performed by ultrasounds (SFX150 Branson, Milan, Italy) for 1 min, followed by homogenization by Ultra-Turrax (T-25 basic, Ika Labortechnik, Staufen im Breisgau, Germany) at 24,000 rpm for 1.5 min and sonication for 1 min. The obtained oil-in-water emulsion was cooled in an ice bath under magnetic stirring for 15 min and then purified by dialysis membrane (MWCO 12–14,000 Da) for 1 h in 300 mL Milli-Q water.

The same procedure was used to prepare unloaded SLNs (U-SLNs). Labeled SLNs for the cell internalization study were obtained by adding 0.01% *w*/*w* Nile Red in the melted stearic acid and following the same method described above for both unloaded (U-SLNs-NR) and PEA-loaded (PEA-SLNs-NR) SLNs.

### 2.4. Particle Size, Z-Potential, and Morphology

Particle size, expressed as Z-average, polydispersity index (PDI), and Z-potential values were measured in deionized water at a concentration of 0.1 g/L by using a photon correlation spectroscopy (PCS) instrument (Zetasizer version 6.12, Malvern Instruments, Worcestershire, UK) equipped with a 4 mW He Ne laser (633 nm) and a DTS software (Version 5.0) [33,34,35]. The reported values averaged determinations on three batches. The morphology of SLNs was evaluated by Atomic Force Microscopy (AFM) (Park Autoprobe Atomic Force Microscope, Park Instruments, Sunnyvale, CA, USA); the measurements were conducted in deionized water at 20 °C and atmospheric pressure operating in a non-contact mode. Freshly prepared samples were water-diluted 1:200 (*v*/*v*) and settled on 1 cm diameter mica disks. After 3 min, the excess water was removed using a paper filter. The images were obtained processing the topographic images by Gwyddion (2.5 version) software.

### 2.5. SLNs Stability

The physical stability of U-SLNs and PEA-SLNs stored at 4 °C was evaluated by monitoring over time (6 months) size, PDI, and Z-potential variations by PCS, as described in Section 2.4. The reported values averaged determinations on three batches.

### 2.6. Drug Loading and Encapsulation Efficiency

PEA loading level (drug loading, DL), i.e., the percentage amount of PEA in SLNs, was assessed by high performance liquid chromatography (HPLC) analysis. The system consisted of two PU-2080 Plus pumps, an HG-980-30 solvent mixing module, and a UV-2075 Plus UV-Vis detector; data were recorded and processed by Hercule Lite Chromatography Interface and Borwin Software, respectively (Jasco Corporation, Tokyo, Japan). Chromatographic separation was performed using a reversed-phase column (RP-18e, 125 × 4 mm, 5.0 μm) thermostatted at 30 °C and protected by a guard column (4.0 × 4.0 mm, 5.0 μm) (Purospher, Merck, Darmstadt, Germany). The mobile phase consisted of acetonitrile (80%) and water (20%) using an isocratic elution with a flow rate of 1 mL/min. The injection volume was 10.0 µL and the UV detection was carried out at 210 nm. Under these experimental conditions, the retention time of PEA was 8.22 ± 0.90 min.

For the HPLC analysis of PEA loaded in SLNs, 10 mL ethanol was added to 700 µL aliquot of PEA-SLNs suspension, heated at 85 °C for 10 min, and filtered through a 0.2 µm filter (17845Q filters, Sartorius, Goettingen, Germany).

Drug loading (DL) and encapsulation efficiency (EE) values, averaged on three batches of SLNs and labeled SLNs, were calculated by using the following equations:

DL% = encapsulated drug (mg)/total mass of SLNs (lipids and drug) × 100

EE% = encapsulated drug (mg)/initial drug (mg) × 100

### 2.7. In Vitro PEA Release

In order to analyze in vitro PEA release from SLNs and PEA dissolution, 500 µL of freshly prepared PEA-SLNs suspension (containing about 1.4 mg of PEA) or the same amount of PEA in bulk were added into 50 mL of PBS and incubated under magnetic stirring at 37 ± 1 °C. At fixed time intervals (30 min, 1 h, 3 h, 6 h, 8 h, and 24 h), an aliquot of the SLNs colloidal suspension or of the supernatant for PEA suspension (1 mL) was withdrawn and filtered through a 0.2 µm filter. The drug content was determined by HPLC using the method mentioned in the Section 2.6. After each withdrawal, 1 mL of fresh PBS was added to the suspension under magnetic stirring in order to re-establish the final volume. Nile Red release from PEA-SLNs-NR was monitored by spectrophotometric quantification (Lambda 3B, Perkin-Elmer, Norwalk, CT, USA) at a wavelength of 553 nm following the same method described above. The analyses were performed in triplicate.

### 2.8. Thermal Analysis

Thermal analyses (thermogram; Tm: melting temperature; ΔHm: melting enthalpy) on PEA-SLNs were performed by differential scanning calorimetry (DSC) (DSC-4, Perkin-Elmer, Norwalk, CT, USA) to investigate the effect of the emulsification preparation technique on the physical state of the components in SLNs lipid matrix. For comparison, thermal analyses were also carried out on a lipid blend/PEA physical mixture, stearic acid/PEA physical mixture, cholesteryl stearate/PEA physical mixture, and each component in bulk. The DSC instrument was previously calibrated with indium. A heating rate of 10 °C/min was employed over a temperature range of 30–120 °C with nitrogen purging (30 mL/min). Analyses were performed in triplicate.

### 2.9. Cell Culture

The C2C12 muscle cell line, derived from satellite cells of the thigh muscle [37], is an immortal line of skeletal myoblasts. C2C12 cells were maintained at low confluence in high glucose DMEM containing L-Glutamine (2 mM), antibiotics (1% P/S), and 10% FBS at 37 °C in a humidified 5% CO_2_ atmosphere.

### 2.10. Cytotoxicity Test

The assay was performed to evaluate the cytotoxicity of PEA-SLNs in comparison with the U-SLNs [34,35]. In practice, cells were seeded in 24-well plates at a density of 4000 cells/well in complete medium for 24 h at 37 °C with 5% CO_2_. Cells were then incubated with different nanoparticle doses (50, 100, or 200 µg/mL) for 24 h. The viability assay was performed 3 times (3 independent experiments) and, for each assay, each sample was tested in experimental triplicate (three wells of a 24-well plate for each sample). After incubation, 3-(4,5 di-methylthiazol-2-yl)-2,5-diphenyltetrazolium bromide (MTT, Sigma Aldrich, Milan, Italy) solution (5 mg/mL in PBS) was added in each well to a final concentration of 0.5 mg/mL. After 2 h, the medium was removed, the cells were washed with PBS and 250 µL MTT solvent (4 mM HCl, 0.1% Igepal in isopropanol) was added to each well to dissolve formazan crystals. After 15 min, the optical densities were measured spectrophometrically at the wavelength of 570 nm (Multiskan™ FC Microplate Photometer, Thermo Fischer Scientific, Monza, Italy). The cell viability was expressed as a percentage of cell growth compared to the control (untreated cells).

### 2.11. Cell Internalization by Flow Cytometry

The capacity of U-SLNs-NR and PEA-SLNs-NR to be internalized by myoblasts was assayed on the C2C12 muscle cell line in comparison with untreated cells as control. To this purpose, the cells were seeded at a density of 80,000 cells/well in 6-wells and incubated with the sample suspensions (200 µg/mL) for 2, 6, and 14 h (overnight). Cells were then washed twice with PBS, detached with 0.25% trypsin-ethylenediaminetetraacetic acid, collected with DMEM, and centrifuged at 1100 rpm for 8 min at 25 °C. Subsequently, the pellets were re-suspended in PBS and analyzed by flow-cytometry using a Coulter Epics XL flow cytometer (Beckman Coulter Inc., Brea, CA, USA) equipped with Ar laser (525 nm). Analyses were performed after recording at least 10,000 events for each sample. Results were expressed as percentages of fluorescence-positive cells.

### 2.12. Confocal Analysis

For confocal analysis, C2C12 cells were seeded at a density of 1500 cells in Nunc Lab-Tek chamber slide (0.7 cm^2^ surface area, Thermo Scientific, Waltham, MA, USA). Cells were treated with 200 µg/mL of U-SLNs-NR, PEA-SLNs-NR, and PEA-SLNs as control; at 14 h post treatment, cells were fixed in paraformaldehyde (3%, *w*/*v*) for 15 min at room temperature, washed in PBS, and observed by microscope. In some cases, the fixed cells in the chamber slide were treated with DAPI to highlight the nuclei. Z-stack images were acquired using a 63× Plan-Apo oil immersion objective mounted on a Leica SP8 confocal microscope (DMIRE2, Leica Microsystems GmbH, Wetzlar, Germany), equipped with 405 nm and white light lasers. Samples were excited using the 405 nm and 668 nm wavelengths for DAPI and Nile Red, respectively.

### 2.13. Statistical Analysis

Data obtained were evaluated statistically using one-way analysis of variance (ANOVA). Significance was indicated by *p* < 0.05 (* *p* < 0.05; ** *p* < 0.02; *** *p* < 0.01).

## 3. Results and Discussion

SLNs were selected as suitable carriers for PEA delivery due to their countless advantages. Indeed, SLNs exhibited a great biocompatibility, biodegradability, and safety regardless of the administration route. Furthermore, SLNs showed a higher physical stability compared with other lipid-based nanoparticles, combined with a high payload of water-insoluble drugs and gradual drug release from the lipid matrix. Finally, the production of SLN might be scalable to large-scale processes without the employment of organic solvents and the final formulations might be sterilized without compromising their features [28,38]. Moreover, lipophilic drugs may remain in the dissolved state within lipid-based drug delivery systems before being absorbed resulting in an increased bioavailability [39].

The treatment of sarcopenia, a whole-body disease, requires a systemic administration. Despite the oral route being the preferred administration pathway due to patient compliance and cost-effectiveness, nanoparticle functions are generally impaired by the biological absorptive barriers of the gastrointestinal tract [28].

As a result, parenteral therapy via an intravenous route is the most effective, especially in the case of lipid-based nanocarriers.

### 3.1. Selection of Formulation Parameters

SLNs formulation was produced without organic solvent by double emulsion/melt dispersion technique described in our previous papers [33,34,35]. The composition selected for SLNs was the result of a pre-formulation investigation using different biocompatible lipids, alone or as blends, and surfactants. Lipids were selected on the basis of their highest apolarity in order to increase solvent property for the strongly lipophilic PEA (log P > 5) [40] and contribute to the solid solution model. Compared to other lipids indeed, stearic acid showed a significant solvent property for PEA at the preparation temperature of 85 °C (520 ± 20 mg/g), presumably due to a certain stereochemical affinity between the fatty acid and PEA, thus improving its spatial arrangement within the lipid chains. For the optimization study, evidence such as SLNs’ smaller size, formation of aggregates, and good PEA encapsulation efficiency were considered. A binary mixture of stearic acid/cholesteryl stearate in a 4:3 ratio was then selected as the lipid matrix for SLNs. This blend maintained a PEA solubility similar to that determined in stearic acid (500 ± 30 mg/g), despite the non-solvent property of cholesteryl stearate towards PEA. From a toxicological point of view, stearic acid and stearic ester of cholesterol represent natural components of the human body; they also proved to be bio-safe for parenteral administration and resulted in a high and prolonged cell survival upon incubation with various types of cells [41]. Span 85 and Pluronic F68 are surfactants of Generally Recognized As Safe (GRAS) status to formulate parenterally administered SLNs. Moreover, Pluronic F68 on the nanoparticle surface may also reduce the reticuloendothelial system clearance [42,43]. The lowest surfactant concentrations combined with the selection of the proper stirring conditions during SLNs preparation process provided the optimized SLNs in terms of size parameters in the pre-formulation study.

### 3.2. SLNs Characterization

The U-SLNs and PEA-SLNs samples were analyzed in terms of size, PDI, and Z-potential values (Table 1).

Indeed, these nanoparticle features are crucial parameters with regard to physical stability, suitability for parenteral administration, bio-distribution, drug release, and cellular uptake [44,45]. The size of SLNs was about 250 nm with a narrow distribution range, as highlighted by the low PDI values. Such a dimensional characteristic is known for being proper to avoid blood clotting and aggregation, including inside fine capillaries, following parenteral administration [43,46]. Similar sub-micron sized carriers were also pointed out for their ability to be taken up by myoblast cells, albeit in a small number of studies from the literature [7]. No substantial differences in size and PDI values were provided by PEA and Nile Red entrapment within the nanoparticles (*p* > 0.05).

**Figure 1:**
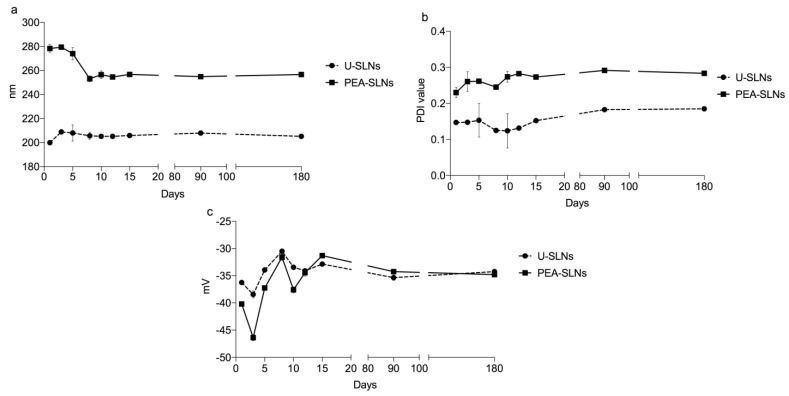
Physical stability of U-SLNs and PEA-SLNs determined by monitoring size (**a**), PDI (**b**), and Z-potential (**c**) values over time.

The Z-potential values of both U-SLNs and PEA-SLNs evidenced a fairly high negative surface charge, without remarkable differences between the samples, owing to the presence of the lipid and surfactant components bearing negative charges. In general, charged surfaces (Z-potential > |20|) are known to prevent particle aggregation in combination with the same effect offered by the steric arrangement of Pluronic molecules at the surface. Furthermore, the negative surface charge is a favorable property since it provides lower cytotoxicity compared to positive or neutral particles and promotes a nanoparticle internalization process by non-phagocytic cells better than the uncharged surfaces [47,48,49,50]. Size, size distribution, and Z-potential values of SLNs did not change significantly for almost 6 months demonstrating the good physical stability of the nanocarrier during the storage (Figure 1).

**Figure 2:**
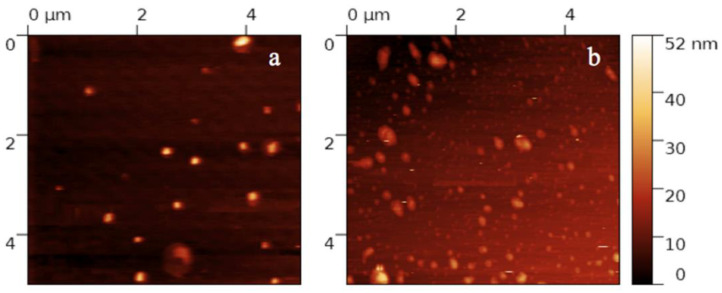
AFM topographic image 5 × 5 µm of U-SLNs (**a**) and PEA-SLNs (**b**).

The morphological analysis carried out on both U-SLNs and PEA-SLNs by AFM revealed distinct nanoparticles with spheroidal or almost elongated shape. In addition, SLNs appeared uniform in shape and size (around 200 nm) and no appreciable differences were detected between U-SLNs and PEA-SLNs (Figure 2).

In addition to appropriate physical properties, particulate carriers require sufficient drug payload to achieve the desired therapeutic effect. From this point of view, lipid matrices are the most suitable carrier structures for embedding highly lipophilic drugs such as PEA in order to favor its molecular dispersion in the perspective of PEA biological actions inside myoblasts. The SLNs’ drug loading level was 16.21 ± 0.3% (*w*/*w*) with an encapsulation efficiency of 91.9 ± 1.0%, regardless of the presence of Nile Red, indicating that PEA was successfully incorporated within SLNs with a poor leaching out from the lipid matrix during the preparation process.

**Figure 3:**
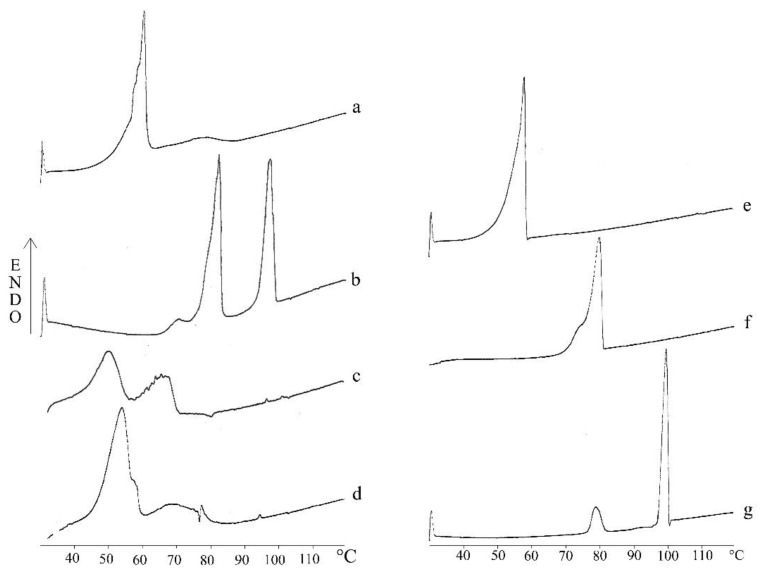
DSC thermograms of stearic acid/PEA physical mixture (**a**), cholesteryl stearate/PEA physical mixture (**b**), PEA-SLNs (**c**), lipid blend/PEA physical mixture (**d**), stearic acid in bulk (**e**), cholesteryl stearate in bulk (**f**), and PEA in bulk (**g**).

Concerning the physical state of PEA and lipid components within SLNs, thermogram recorded for PEA-SLNs was compared with those obtained for physical mixtures at the same ratio as that of nanoparticles, i.e., stearic acid/PEA physical mixture, cholesteryl stearate/PEA physical mixture, and lipid blend/PEA physical mixture, and for each component in bulk (Figure 3).

Thermogram of PEA-SLNs exhibited an endothermic event at 50.6 °C referable to the melting of stearic acid (Tm). The shift towards lower Tm value and the peak broadening compared with the fatty acid in bulk (Tm 57.9 °C) and in the lipid blend/PEA physical mixture (Tm 54.9 °C) may be attributed to a certain disorder in the crystal structure provided by the binary mixture with cholesteryl stearate [33,51]. Likewise, a less-ordered structure was also observed for cholesteryl stearate within the PEA-SLNs matrix, as demonstrated by Tm shift to a lower value (68.1 °C) than that of the lipid in bulk (Tm 80.0 °C) and in the physical mixture (Tm 78 °C). The slightly pronounced endotherm at 95.3 °C (ΔHm 0.88 J/g) recorded for PEA-SLNs and for lipid blend/PEA physical mixture (Tm 97.3 °C, ΔHm 1.26 J/g) can be attributed to PEA (Tm 99.8 °C) even if bulk PEA exhibited a significantly higher ΔH value (ΔHm 190.92 J/g) [31,52]. The relevant decrease of PEA melting enthalpy observed in both PEA-SLNs and lipid blend/PEA physical mixture with respect to bulk PEA indicates that PEA is essentially embedded inside the lipid matrix in an amorphous state. This finding is consistent with PEA solubility in the lipid blend containing stearic acid that acts as a solvent in its melted state during both SLNs preparation and DSC run. The molecular dispersion state of PEA attributed to stearic acid component was confirmed by the behavior of PEA mixed with each lipid component at the same ratio as that in SLNs. In fact, thermal events ascribable to PEA were not recorded in stearic acid/PEA physical mixture, indicating the formation of a solid solution. In contrast, a PEA thermal event with melting enthalpy similar to that of bulk PEA was observed in cholesteryl stearate/PEA physical mixture (ΔHm 161.19 J/g), thus evidencing both PEA crystalline state and non-solvent property of this lipid component.

**Figure 4:**
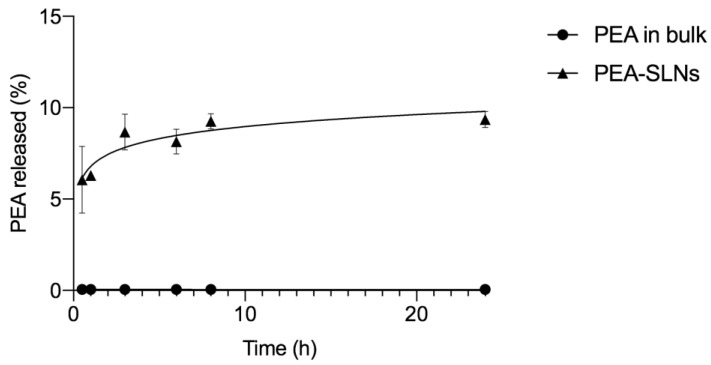
Release of PEA from PEA-SLNs and dissolution of PEA in bulk using PBS as medium. Results are presented as the average ± SD (*n* = 3).

The goal to reach muscle tissues by systemic administration of PEA-SLNs also implies the prediction of the drug leaching out from the nanoparticles before their internalization by myoblasts occurs. For this reason, in vitro release of PEA from SLNs was investigated in comparison with the dissolution of PEA in bulk (Figure 4).

The release pattern of PEA from PEA-SLNs exhibits a biphasic trend. The initial rapid release, 6% in 2 h, was followed by a gradual release reaching 9% of the drug loaded in 24 h. The initial release should be ascribed to the presence in the outermost part of the lipid matrix of amorphous drug that is more soluble relative to the practically insoluble crystalline PEA in bulk. Indeed, no dissolution of PEA in bulk was observed during 24 h in the adopted experimental conditions (*p* < 0.05). The second sustained release phase may be due to the extended leakage of the amorphous drug entrapped in the inner core of lipid matrix. It can be assumed that the considerable portion of residual drug into SLNs may be released by lipolytic processes that take place within the cells in vivo, i.e., fatty acid oxidation inside mitochondria and cholesteryl ester hydrolysis inside endosomes [53,54].

No Nile Red release was also observed from PEA-SLNs-NR in the same experimental conditions. In accordance with this result, firm and prolonged incorporation of Nile Red in several lipid structures, with minimal leakage effects even without covalent linkages, was also found by other authors and associated to the highly hydrophobic nature of this dye [55,56,57].

### 3.3. Cell Culture Assay

As regards the drug delivery through nanocarriers, it has been generally observed that nanoparticles taken up by myoblasts promoted the formation of myotubes that constitute the skeletal muscle by enhancing myoblast fusion [7]. This goal would imply that quite intact nanoparticles reach the targeted sites without an appreciable drug loading loss in physiological environment, i.e., before internalization by myoblast occurs. This latter feature was confirmed for PEA-SLNs from in vitro drug release data. Concerning the biodegradation of lipid-based vehicles in the blood stream, an increase of drug bioavailability was observed following intravenous administration owing to the prolonged systemic circulation of the lipid vehicles compared with the pure drug [58]. Biodegradation of SLNs, which occurs mainly by serum lipases, is not expected for the SLNs presented in this study thanks to their lipid composition and sterically hindering surfactant [59]. From plasma, SLNs have to enter into skeletal muscle myoblasts where lipids will be subjected to degradation by lipolytic processes, as mentioned above. Therefore, to optimize intracellular PEA release and activity, it is mandatory to investigate the interaction between SLNs and skeletal muscle cells in terms of cytotoxicity and internalization capacity. Studies involving the behavior of lipid-based nanocarriers upon contact with skeletal muscle cells has been poorly reported in the literature and mainly focused on liposomes [7,60,61].

**Figure 5:**
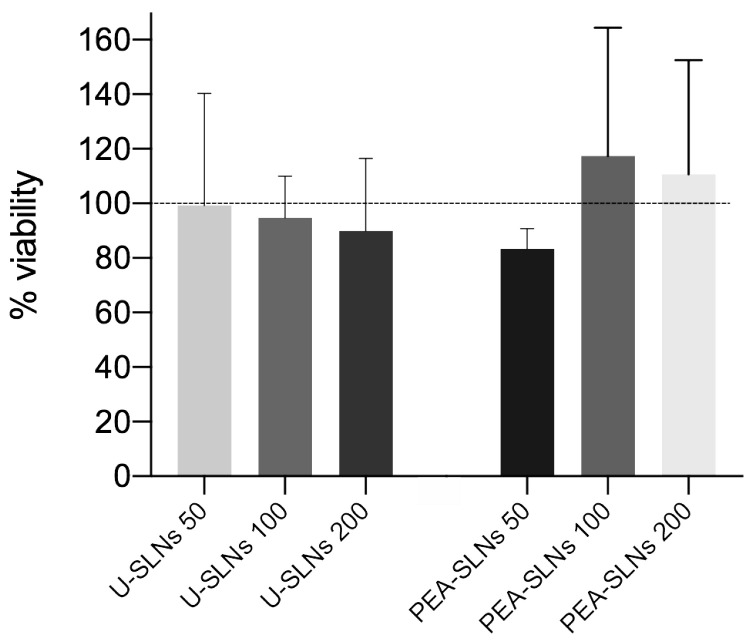
Cytotoxicity of U-SLNs and PEA-SLNs at the concentrations of 50, 100, and 200 µg/mL on C2C12 cell line at 24 h incubation, expressed as cell viability percentage (dotted line as the control).

The commonly used C2C12 muscle cell line was employed for the assessment of both cytotoxicity and cell internalization. As regards cytotoxicity, Figure 5 shows MTT results obtained for U-SLNs and PEA-SLNs samples in comparison with untreated control cells, expressed as cell viability in function of dose. Considering that nanoparticle cytotoxicity is related to cell internalization also requiring several hours in the case of myoblasts [62], C2C12 cells were exposed to SLNs sample for 24 h.

Cell incubation with U-SLNs maintained viability levels above 90%, regardless of the dose (*p* > 0.05). We noticed a positive effect of PEA since cytotoxicity of PEA-SLNs has a tendency to decrease with the increase of SLNs dose from 50 µg/mL to 100 µg/mL and cell viability for PEA-SLNs doses of both 100 and 200 µg/mL is higher compared to unloaded SLNs. This finding may indicate, on one side, the non-toxic effect of PEA, and on the other, PEA ability to restore the cell susceptibility. This event, which also induces cell activation, was observed by other authors on neuronal cells and attributed to PEA antioxidant properties [63].

The investigation of SLNs’ internalization by C2C12 cell line was performed on U-SLNs-NR and PEA-SLNs-NR by means of flow cytometry and confocal microscopy. The cells were exposed to a SLNs dose of 200 µg/mL, regardless of the sample. SLNs labeling was related to the firm entrapment of Nile Red, recognized to be an excellent fluorescent lipid marker, within the lipid matrix. This assumption arises from the in vitro release studies that demonstrated Nile Red inability to be released from SLNs into PBS [55,56].

**Figure 6:**
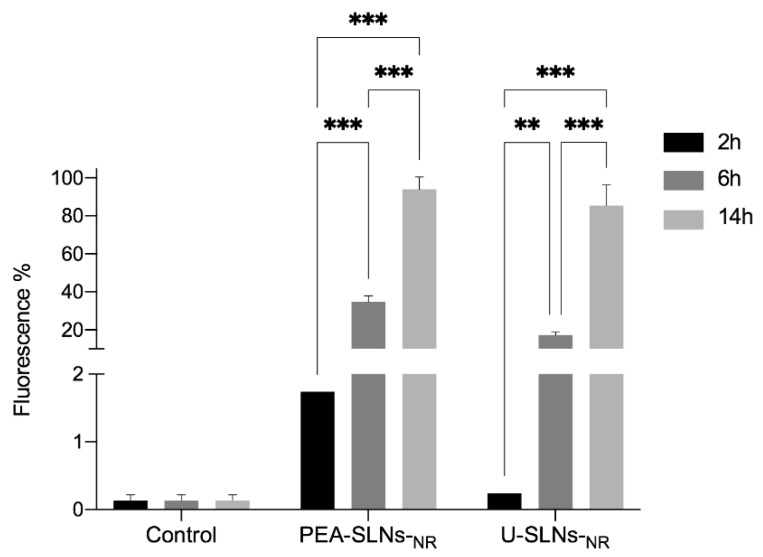
Flow cytometric analysis of C2C12 cells after 2, 6, and 14 h incubation with PEA-SLNs-NR and U-SLNs-NR in comparison with untreated cells (control). For the points at 2 h, error lines do not exceed bar size. Statistical significance levels are indicated as: ** (*p* < 0.02); *** (*p* < 0.01).

The results of the flow cytometric analysis at different incubation times, expressed as the mean cell fluorescence percentage, are shown in Figure 6.

The cells incubated with unlabeled PEA-SLNs exhibited negligible fluorescence emission, regardless of the incubation time, comparable to that from the control (untreated cells) (from 0.1 to 0.2% fluorescence), which indicates the absence of auto-fluorescence phenomena. Both the labeled samples provided a significant time-dependent increase of the mean cell fluorescence percentage (*p* < 0.02 or 0.01), reaching values between 85 and 94% at 14 h incubation.

**Figure 7:**
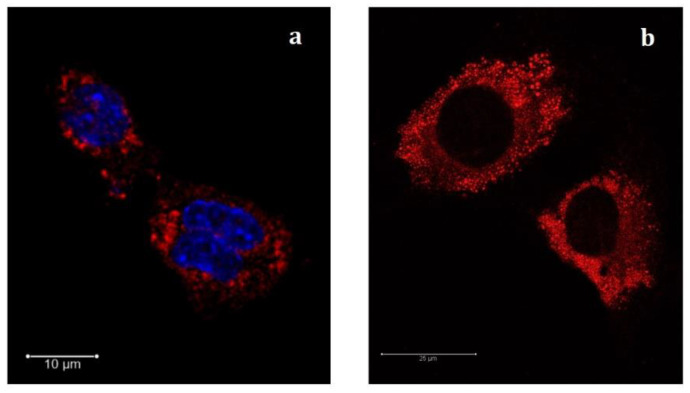
Representative confocal fluorescence images of C2C12 cells exposed to PEA-SLNs-NR (red) during 14 h. SLNs (red channel) and nucleus (blue channel) were stained with Nile Red and DAPI, respectively (**a**); image obtained with no nuclear stain (**b**).

As a matter of fact, the fluorescence measured by flow cytometry could be due to the presence of SLNs both at the surface and inside the cytoplasm; therefore, further analysis by confocal microscopy was performed. Confocal microscopy images of cells subjected to fluorescently labeled U-SLNs-NR and PEA-SLNs-NR (200 µg/mL) were used to provide visual confirmation of cell internalization of both SLNs. Similar results were obtained for the two formulations and only images related to PEA-SLNs-NR after 14 h incubation are presented in Figure 7. DAPI was used as nucleus stain (blue) to provide a visual location of cells (Figure 7a).

Marked red spots were observed in the cytoplasmatic perinuclear region upon cell exposure to PEA-SLNs-NR. The red punctuated staining is reasonably related to the intracellular presence of nanoparticles also in the form of aggregates or stored in subcellular locations [54]. This finding, complementing the results from the flow cytometric analysis, demonstrates the capacity of SLNs to be internalized by murine C2C12 myoblast cells. Myoblast capacity to take up nanoparticles was demonstrated by few authors and was found to be related to the nanoparticle type. It was described as an endocytic process, which lasts several hours after cell exposure, involving mainly mechanisms of macropinocyte and clathrin-mediated pathway [7]. As can be observed in the image obtained without staining the nucleus (Figure 7b), no trace of fluorescence is visible in the nucleus or in the nucleoli. This finding demonstrates, on one hand, that the red fluorescence is attributable unequivocally to the uptake of labeled nanoparticles and not to the passive diffusion of the marker; indeed, if a non-specific diffusion of the Nile Red had occurred, this would be observed not only in the cytoplasm but also in the nucleus. On the other hand, the presence of red spots only in the cytoplasm demonstrates that SLNs are unable to cross the nuclear membrane. However, considering the subsequent intracellular biodegradation of the lipid matrix, the leaching out of PEA from SLNs can be envisaged as well as its gaining cytoplasmatic and nuclear receptors as PPAR-α, thus exerting its pharmacological activity inside myoblasts.

Although our data are encouraging, it is important to consider that in vivo adult muscle is made up of multinucleated cells which are embedded within a complex three-dimensional scaffold and the skeletal muscle extracellular matrix might limit the ability of nanoparticles to deliver PEA; therefore, the next step will be to test absorption in vivo in an animal mouse model. Moreover, a further goal is to clarify whether the administration of PEA via nanoparticles is able to counteract the negative effects of TNF- α on C2C12 terminal differentiation. These studies are essential to begin the path towards the commercial availability of a safe therapy for sarcopenia which can represent a tool without side effects in clinical practice.

## 4. Conclusions

Aging of skeletal muscle as a target of therapeutic strategies is particularly attractive due to both the high incidence of related dysfunctions and to the limited literature available. In this scenario, the present investigation, focused on PEA use and interactions of SLNs embedding PEA with skeletal muscle cells, has provided novel data. Our findings on PEA-SLNs confirm the absence of cytotoxicity and the ability to be taken up by myoblast cell line and suggest the potentiality provided by the lipid nanocarrier in sarcopenia treatment which can represent a safe tool without side effects in clinical practice.

## Figures and Tables

**Table 1:** Size, PDI, and Z-potential values of U-SLNs and PEA-SLNs. Mean values ± SD.

Sample	Size (nm)	PDI	Z Potential (mV)
U-SLNs	214 ± 19	0.244 ± 0.050	−32.3 ± 2.5
PEA-SLNs	253 ± 15	0.185 ± 0.100	−39.5 ± 2.1
PEA-SLNs-NR	258 ± 12	0.197 ± 0.080	−40.2 ± 2.4

## References

[1] Cruz-Jentoft A.J., Bahat G., Bauer J., Boirie Y., Bruyère O., Cederholm T., Cooper C., Landi F., Rolland Y., Sayer A.A. (2019). Sarcopenia: Revised European Consensus on Definition and Diagnosis. Age Ageing.

[2] Balogun S., Winzenberg T., Wills K., Scott D., Jones G., Callisaya M.L., Aitken D. (2019). Prospective Associations of Low Muscle Mass and Strength with Health-Related Quality of Life over 10-Year in Community-Dwelling Older Adults. Exp. Gerontol..

[3] Morley J.E. (2018). Treatment of Sarcopenia: The Road to the Future. J. Cachexia Sarcopenia Muscle.

[4] Giles J.T., Ling S.M., Ferrucci L., Bartlett S.J., Andersen R.E., Towns M., Muller D., Fontaine K.R., Bathon J.M. (2008). Abnormal Body Composition Phenotypes in Older Rheumatoid Arthritis Patients: Association with Disease Characteristics and Pharmacotherapies. Arthritis Rheum..

[5] Lo J.H.-T., Yiu T., Ong M.T.-Y., Lee W.Y.-W. (2020). Sarcopenia: Current Treatments and New Regenerative Therapeutic Approaches. J. Orthop. Translat..

[6] Kwak J.Y., Kwon K.-S. (2019). Pharmacological Interventions for Treatment of Sarcopenia: Current Status of Drug Development for Sarcopenia. Ann. Geriatr. Med. Res..

[7] Guglielmi V., Carton F., Vattemi G., Arpicco S., Stella B., Berlier G., Marengo A., Boschi F., Malatesta M. (2019). Uptake and Intracellular Distribution of Different Types of Nanoparticles in Primary Human Myoblasts and Myotubes. Int. J. Pharm..

[8] Raimondo T.M., Mooney D.J. (2018). Functional Muscle Recovery with Nanoparticle-Directed M2 Macrophage Polarization in Mice. Proc. Natl. Acad. Sci. USA.

[9] Nishikawa H., Fukunishi S., Asai A., Yokohama K., Nishiguchi S., Higuchi K. (2021). Pathophysiology and Mechanisms of Primary Sarcopenia. Int. J. Mol. Med..

[10] Dalle S., Rossmeislova L., Koppo K. (2017). The Role of Inflammation in Age-Related Sarcopenia. Front. Physiol..

[11] Yoo S.-Z., No M.-H., Heo J.-W., Park D.-H., Kang J.-H., Kim S.H., Kwak H.-B. (2018). Role of Exercise in Age-Related Sarcopenia. J. Exerc. Rehabil..

[12] Perna S., Alalwan T.A., Al-Thawadi S., Negro M., Parimbelli M., Cerullo G., Gasparri C., Guerriero F., Infantino V., Diana M. (2020). Evidence-Based Role of Nutrients and Antioxidants for Chronic Pain Management in Musculoskeletal Frailty and Sarcopenia in Aging. Geriatrics.

[13] Landi F., Marzetti E., Liperoti R., Pahor M., Russo A., Martone A.M., Colloca G., Capoluongo E., Bernabei R. (2013). Nonsteroidal Anti-Inflammatory Drug (NSAID) Use and Sarcopenia in Older People: Results from the IlSIRENTE Study. J. Am. Med. Dir. Assoc..

[14] Trappe T.A., Ratchford S.M., Brower B.E., Liu S.Z., Lavin K.M., Carroll C.C., Jemiolo B., Trappe S.W. (2016). COX Inhibitor Influence on Skeletal Muscle Fiber Size and Metabolic Adaptations to Resistance Exercise in Older Adults. J. Gerontol. A Biol. Sci. Med. Sci..

[15] Uchitomi R., Oyabu M., Kamei Y. (2020). Vitamin D and Sarcopenia: Potential of Vitamin D Supplementation in Sarcopenia Prevention and Treatment. Nutrients.

[16] Keppel Hesselink J.M., de Boer T., Witkamp R.F. (2013). Palmitoylethanolamide: A Natural Body-Own Anti-Inflammatory Agent, Effective and Safe against Influenza and Common Cold. Int. J. Inflam..

[17] Uberti F., Ruga S., Farghali M., Galla R., Molinari C. (2021). A Combination of α-Lipoic Acid (ALA) and Palmitoylethanolamide (PEA) Blocks Endotoxin-Induced Oxidative Stress and Cytokine Storm: A Possible Intervention for COVID-19. J. Diet. Suppl..

[18] Gabrielsson L., Gouveia-Figueira S., Häggström J., Alhouayek M., Fowler C.J. (2017). The Anti-Inflammatory Compound Palmitoylethanolamide Inhibits Prostaglandin and Hydroxyeicosatetraenoic Acid Production by a Macrophage Cell Line. Pharmacol. Res. Perspect..

[19] Heide E.C., Bindila L., Post J.M., Malzahn D., Lutz B., Seele J., Nau R., Ribes S. (2018). Prophylactic Palmitoylethanolamide Prolongs Survival and Decreases Detrimental Inflammation in Aged Mice with Bacterial Meningitis. Front. Immunol..

[20] Orefice N.S., Alhouayek M., Carotenuto A., Montella S., Barbato F., Comelli A., Calignano A., Muccioli G.G., Orefice G. (2016). Oral Palmitoylethanolamide Treatment Is Associated with Reduced Cutaneous Adverse Effects of Interferon-Β1a and Circulating Proinflammatory Cytokines in Relapsing–Remitting Multiple Sclerosis. Neurotherapeutics.

[21] Peritore A.F., Siracusa R., Fusco R., Gugliandolo E., D’Amico R., Cordaro M., Crupi R., Genovese T., Impellizzeri D., Cuzzocrea S. (2020). Ultramicronized Palmitoylethanolamide and Paracetamol, a New Association to Relieve Hyperalgesia and Pain in a Sciatic Nerve Injury Model in Rat. Int. J. Mol. Sci..

[22] Huang K., Masuda A., Chen G., Bushra S., Kamon M., Araki T., Kinoshita M., Ohkawara B., Ito M., Ohno K. (2020). Inhibition of Cyclooxygenase-1 by Nonsteroidal Anti-Inflammatory Drugs Demethylates MeR2 Enhancer and Promotes Mbnl1 Transcription in Myogenic Cells. Sci. Rep..

[23] Mastbergen S.C., Lafeber F.P.J.G., Bijlsma J.W.J. (2002). Selective COX-2 Inhibition Prevents Proinflammatory Cytokine-Induced Cartilage Damage. Rheumatology.

[24] Trappe T.A., Liu S.Z. (1985). Effects of Prostaglandins and COX-Inhibiting Drugs on Skeletal Muscle Adaptations to Exercise. J. Appl. Physiol..

[25] Beggiato S., Tomasini M.C., Ferraro L. (2019). Palmitoylethanolamide (PEA) as a Potential Therapeutic Agent in Alzheimer’s Disease. Front. Pharmacol..

[26] Cordaro M., Cuzzocrea S., Crupi R. (2020). An Update of Palmitoylethanolamide and Luteolin Effects in Preclinical and Clinical Studies of Neuroinflammatory Events. Antioxidants.

[27] Peritore A.F., Siracusa R., Crupi R., Cuzzocrea S. (2019). Therapeutic Efficacy of Palmitoylethanolamide and Its New Formulations in Synergy with Different Antioxidant Molecules Present in Diets. Nutrients.

[28] Mitchell M.J., Billingsley M.M., Haley R.M., Wechsler M.E., Peppas N.A., Langer R. (2021). Engineering Precision Nanoparticles for Drug Delivery. Nat. Rev. Drug Discov..

[29] Patra J.K., Das G., Fraceto L.F., Campos E.V.R., Rodriguez-Torres M.d.P., Acosta-Torres L.S., Diaz-Torres L.A., Grillo R., Swamy M.K., Sharma S. (2018). Nano Based Drug Delivery Systems: Recent Developments and Future Prospects. J. Nanobiotechnol..

[30] Puglia C., Santonocito D., Ostacolo C., Maria Sommella E., Campiglia P., Carbone C., Drago F., Pignatello R., Bucolo C. (2020). Ocular Formulation Based on Palmitoylethanolamide-Loaded Nanostructured Lipid Carriers: Technological and Pharmacological Profile. Nanomaterials.

[31] Tronino D., Offerta A., Ostacolo C., Russo R., De Caro C., Calignano A., Puglia C., Blasi P. (2016). Nanoparticles Prolong N-Palmitoylethanolamide Anti-Inflammatory and Analgesic Effects in Vivo. Colloids Surf. B Biointerfaces.

[32] Hosny K.M., Sindi A.M., Ali S., Alharbi W.S., Hajjaj M.S., Bukhary H.A., Badr M.Y., Mushtaq R.Y., Murshid S.S.A., Almehmady A.M. (2022). Development, optimization, and evaluation of a nanostructured lipid carrier of sesame oil loaded with miconazole for the treatment of oral candidiasis. Drug Deliv..

[33] Maretti E., Costantino L., Buttini F., Rustichelli C., Leo E., Truzzi E., Iannuccelli V. (2019). Newly Synthesized Surfactants for Surface Mannosylation of Respirable SLN Assemblies to Target Macrophages in Tuberculosis Therapy. Drug Deliv. Transl. Res..

[34] Maretti E., Rustichelli C., Romagnoli M., Balducci A.G., Buttini F., Sacchetti F., Leo E., Iannuccelli V. (2016). Solid Lipid Nanoparticle Assemblies (SLNas) for an Anti-TB Inhalation Treatment—A Design of Experiments Approach to Investigate the Influence of Pre-Freezing Conditions on the Powder Respirability. Int. J. Pharm..

[35] Sacchetti F., Marraccini C., D’Arca D., Pelà M., Pinetti D., Maretti E., Hanuskova M., Iannuccelli V., Costi M.P., Leo E. (2015). Enhanced Anti-Hyperproliferative Activity of Human Thymidylate Synthase Inhibitor Peptide by Solid Lipid Nanoparticle Delivery. Colloids Surf. B.

[36] Kurakula M., Ahmed O.A.A., Fahmy U.A., Ahmed T.A. (2016). Solid lipid nanoparticles for transdermal delivery of avanafil: Optimization, formulation, in-vitro and ex-vivo studies. J. Liposome Res..

[37] Yaffe D., Saxel O. (1977). Serial Passaging and Differentiation of Myogenic Cells Isolated from Dystrophic Mouse Muscle. Nature.

[38] Tenchov R., Bird R., Curtze A.E., Zhou Q. (2021). Lipid Nanoparticles—From Liposomes to MRNA Vaccine Delivery, a Landscape of Research Diversity and Advancement. ACS Nano.

[39] Rane S.S., Anderson B.D. (2008). What Determines Drug Solubility in Lipid Vehicles: Is It Predictable?. Adv. Drug Deliv. Rev..

[40] Lambert D.M., Vandevoorde S., Diependaele G., Govaerts S.J., Robert A.R. (2001). Anticonvulsant Activity of N-Palmitoylethanolamide, a Putative Endocannabinoid, in Mice. Epilepsia.

[41] Vighi E., Montanari M., Hanuskova M., Iannuccelli V., Coppi G., Leo E. (2013). Design Flexibility Influencing the in Vitro Behavior of Cationic SLN as a Nonviral Gene Vector. Int. J. Pharm..

[42] Carrstensen H., Müller R.H., Müller B.W. (1992). Particle Size, Surface Hydrophobicity and Interaction with Serum of Parenteral Fat Emulsions and Model Drug Carriers as Parameters Related to RES Uptake. Clin. Nutr..

[43] Wissing S.A., Kayser O., Müller R.H. (2004). Solid Lipid Nanoparticles for Parenteral Drug Delivery. Adv. Drug Deliv. Rev..

[44] Azhar Shekoufeh Bahari L., Hamishehkar H. (2016). The Impact of Variables on Particle Size of Solid Lipid Nanoparticles and Nanostructured Lipid Carriers. Adv. Pharm. Bull..

[45] Danaei M., Dehghankhold M., Ataei S., Hasanzadeh Davarani F., Javanmard R., Dokhani A., Khorasani S., Mozafari M.R. (2018). Impact of Particle Size and Polydispersity Index on the Clinical Applications of Lipidic Nanocarrier Systems. Pharmaceutics.

[46] Siekmann B., Westesen K. (1992). Submicron-Sized Parenteral Carrier Systems Based on Solid Lipids. Pharm. Pharmacol. Lett..

[47] Bhattacharjee S., de Haan L.H.J., Evers N.M., Jiang X., Marcelis A.T.M., Zuilhof H., Rietjens I.M.C.M., Alink G.M. (2010). Role of Surface Charge and Oxidative Stress in Cytotoxicity of Organic Monolayer-Coated Silicon Nanoparticles towards Macrophage NR8383 Cells. Part. Fibre Toxicol..

[48] Foroozandeh P., Aziz A.A. (2018). Insight into Cellular Uptake and Intracellular Trafficking of Nanoparticles. Nanoscale Res. Lett..

[49] Fröhlich E. (2012). The Role of Surface Charge in Cellular Uptake and Cytotoxicity of Medical Nanoparticles. Int. J. Nanomed..

[50] Kelly C., Jefferies C., Cryan S.-A. (2010). Targeted Liposomal Drug Delivery to Monocytes and Macrophages. J. Drug Deliv..

[51] Severino P., Pinho S.C., Souto E.B., Santana M.H.A. (2011). Polymorphism, Crystallinity and Hydrophilic-Lipophilic Balance of Stearic Acid and Stearic Acid-Capric/Caprylic Triglyceride Matrices for Production of Stable Nanoparticles. Colloids Surf. B Biointerfaces.

[52] Campardelli R., Oleandro E., Scognamiglio M., Della Porta G., Reverchon E. (2017). Palmitoylethanolamide Sub-Micronization Using Fast Precipitation Followed by Supercritical Fluids Extraction. Powder Technol..

[53] Morales P.E., Bucarey J.L., Espinosa A. (2017). Muscle Lipid Metabolism: Role of Lipid Droplets and Perilipins. J. Diabetes Res..

[54] Watt M.J., Hoy A.J. (2012). Lipid Metabolism in Skeletal Muscle: Generation of Adaptive and Maladaptive Intracellular Signals for Cellular Function. Am. J. Physiol. Endocrinol. Metab..

[55] Bouchaala R., Anton N., Anton H., Vandamme T., Vermot J., Smail D., Mély Y., Klymchenko A.S. (2017). Light-Triggered Release from Dye-Loaded Fluorescent Lipid Nanocarriers In Vitro and In Vivo. Colloids Surf. B Biointerfaces.

[56] Greenspan P., Mayer E.P., Fowler S.D. (1985). Nile Red: A Selective Fluorescent Stain for Intracellular Lipid Droplets. J. Cell Biol..

[57] Vighi E., Montanari M., Ruozi B., Tosi G., Magli A., Leo E. (2010). Nuclear Localization of Cationic Solid Lipid Nanoparticles Containing Protamine as Transfection Promoter. Eur. J. Pharm. Biopharm..

[58] Xie S., Zhu L., Dong Z., Wang X., Wang Y., Li X., Zhou W. (2011). Preparation, Characterization and Pharmacokinetics of Enrofloxacin-Loaded Solid Lipid Nanoparticles: Influences of Fatty Acids. Colloids Surf. B.

[59] Olbrich C., Kayser O., Müller R.H. (2002). Lipase Degradation of Dynasan 114 and 116 Solid Lipid Nanoparticles (SLN)—Effect of Surfactants, Storage Time and Crystallinity. Int. J. Pharm..

[60] Katayama T., Kinugawa S., Takada S., Furihata T., Fukushima A., Yokota T., Anzai T., Hibino M., Harashima H., Yamada Y. (2019). A Mitochondrial Delivery System Using Liposome-Based Nanocarriers That Target Myoblast Cells. Mitochondrion.

[61] Mukherjee S., Maity S., Ghosh B., Chakraborty T., Mondal A., Bishayee A. (2020). Assessment of the Antidiabetic Potentiality of Glyburide Loaded Glyceryl Monostearate Solid Lipid Nanoparticles. J. Drug Deliv. Sci. Technol..

[62] Sun H., Jiang C., Wu L., Bai X., Zhai S. (2019). Cytotoxicity-Related Bioeffects Induced by Nanoparticles: The Role of Surface Chemistry. Front. Bioeng. Biotechnol..

[63] Morsanuto V., Galla R., Molinari C., Uberti F. (2020). A New Palmitoylethanolamide Form Combined with Antioxidant Molecules to Improve Its Effectivess on Neuronal Aging. Brain Sci..

